# Diagnostic performance of an automated robot for MALDI target preparation in microbial identification

**DOI:** 10.1128/jcm.00434-24

**Published:** 2024-09-19

**Authors:** Arthur B. Pranada, Michal Cicatka, Clara Heß, Jan Karasek

**Affiliations:** 1 Division of Medical Microbiology, MVZ Dr. Eberhard & Partner Dortmund, Dortmund, Germany; 2 Department of Telecommunication, Faculty of Electrical Engineering and Communications, Brno University of Technology, Brno, Czech Republic; 3 R&D Automation, Microbiology & Diagnostics, Bruker Daltonics GmbH & Co. KG, Bremen, Germany; NorthShore University HealthSystem Department of Pathology and Laboratory Medicine, Evanston, Illinois, USA

**Keywords:** MALDI-TOF, mass spectrometry, colony picking, MBT Pathfinder, automation, sample preparation

## Abstract

The MBT Pathfinder is an automated colony-picking robot designed for efficient sample preparation in matrix-assisted laser desorption/ionization time-of-flight (MALDI-TOF) mass spectrometry. This article presents results from three key experiments evaluating the instrument’s performance in conjunction with MALDI Biotyper instrument. The method comparison experiment assessed its clinical performance, demonstrating comparable results with gram-positive, gram-negative, and anaerobic bacteria (scores larger than 2.00) and superior performance over simple direct yeast transfer (score: 1.80) when compared to samples prepared manually. The repeatability experiment confirmed consistent performance over multiple days and labs (average log score: 2.12, std. deviation: 0.59). The challenge panel experiment showcased its consistent and accurate performance across various samples and settings, yielding average scores between 1.76 and 2.19. These findings underline the MBT Pathfinder as a reliable and efficient tool for MALDI-TOF mass spectrometry sample preparation in clinical and research applications.

## INTRODUCTION

The matrix-assisted laser desorption/ionization time-of-flight (MALDI-TOF) technology has become integral to the operations of numerous biomedical laboratories worldwide (1, 2). It is widely employed for microbial identification and analysis, encompassing bacteria, yeasts, and molds. Sample preparation varies primarily based on both the cultivation media used for microbial analysis and the technique of transferring the microbes onto the analytical sample carriers known as MALDI target plates (3). Some prominent MALDI-TOF systems include MALDI Biotyper and VITEK MS, and their performance across various microbes has been extensively studied (4–6), along with the influence of different sample preparation techniques (7).

In the current landscape of biomedical laboratories, there is a notable trend toward total laboratory automation (TLA) to minimize costs, streamline repetitive tasks typically carried out by skilled laboratory personnel, and facilitate the training of new staff (8). However, the manual preparation of MALDI target plates remains a significant bottleneck for TLA in the context of MALDI-TOF (9). Addressing this issue, colony pickers, a specialized type of robots designed for automated transfer of microbial colonies from agar plates to MALDI target plates (10), have emerged.

Recently, Bruker Daltonics GmbH & Co. KG introduced a prototype of a compact colony-picking robot, MBT Pathfinder. This system functions independently from other TLA instrumentation, ensuring flexible integration into various laboratory workflows. The MBT Pathfinder is also available in a configuration that includes the Feeder, a robotic arm designed for automated manipulation with agar plates, further enhancing its automation capabilities. This study primarily aims to evaluate and compare its performance to the manual workflow in combination with the MALDI Biotyper MALDI-TOF, a well-established reference system in this domain.

## MATERIALS AND METHODS

We designed one clinical and two analytical experiments in order to evaluate the performance of the MBT Pathfinder colony-picking system. The experiments were run in two microbiology routine laboratories in Germany with two operators in each laboratory. The experiments included both manual MALDI target plate sample preparation and automated preparation with the MBT Pathfinder system. Each laboratory worked with a designated MBT Pathfinder and MALDI Biotyper systems. The MALDI Biotyper generates log scores within the range of 0 to 3.0, with identification thresholds for bacteria and yeasts set at log scores of ≥2.0 for an identification at high confidence and ≥1.7 for identification at low confidence level. Log score values <1.7 are regarded as unreliable identification. Aforementioned thresholds are suggested by the user manual of the MALDI Biotyper instrument. These log scores from the mass spectrometry analysis of the prepared MALDI target plates served as the indicators of preparation quality, effectively reflecting the performance of the MBT Pathfinder instrument.

Except for the anaerobic species *Prevotella bivia*, *Clostridium perfringens*, *Bacteroides vulgatus*, *Bacteroides thetaiotaomicron*, and *Bacteroides ovatus*, which were cultured using BD Schaedler Agar with Vitamin K1 and 5% Sheep Blood agar, all other samples were cultivated using BD Columbia Agar with 5% Sheep Blood. In this study, microbial samples were cultured from cryogenic stock on dedicated agar media and incubated for 18–48 h at a cultivation temperature of 36 ± 1°C. Colonies from primary cultures were isolated on dedicated agar plates and incubated again, with second subcultures used the following morning in an experiment.

**FIG 1:**
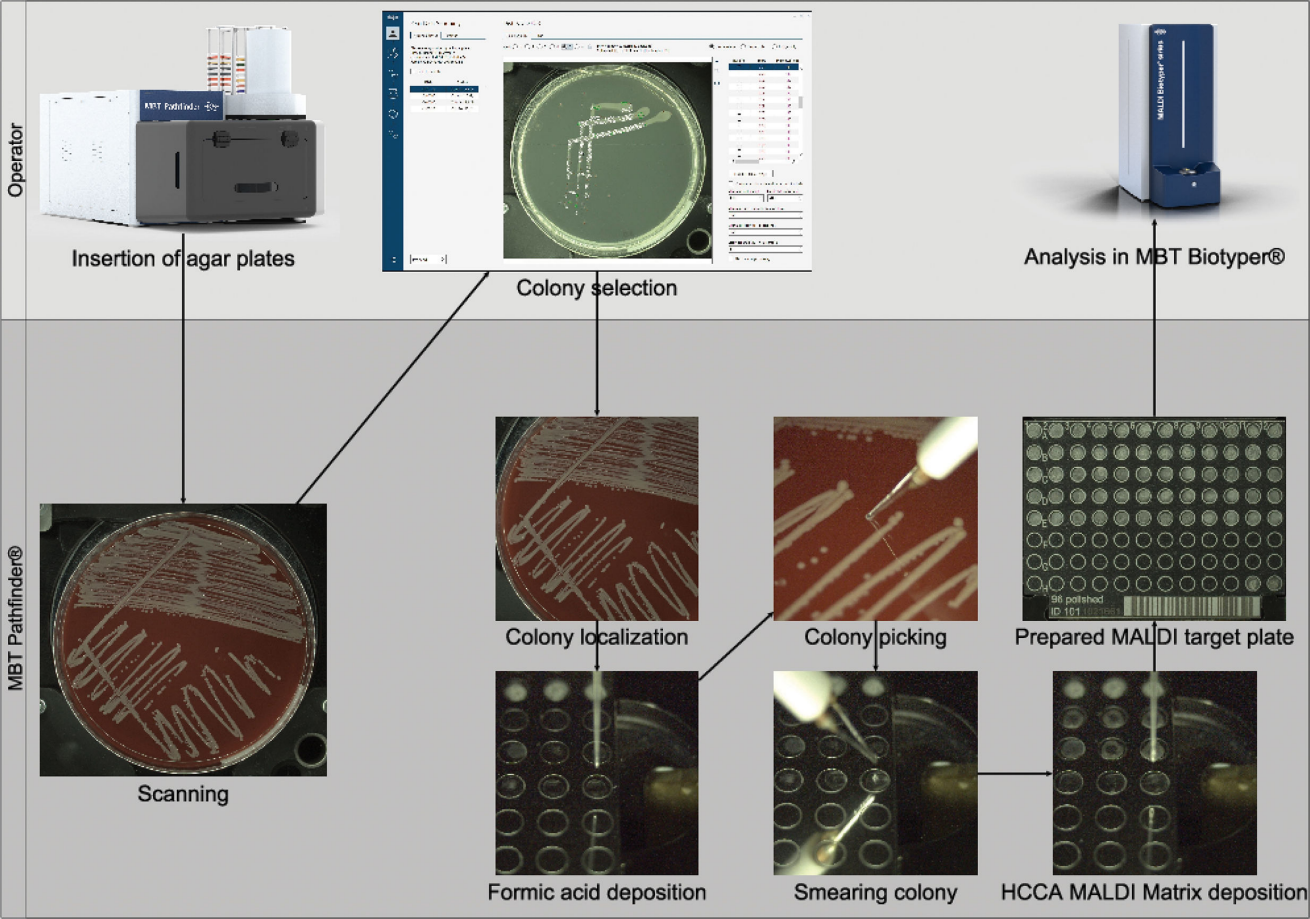
Diagram illustrating the workflow of the reDT method.

The experiments involved two well-established methods for manual sample preparation: Direct Transfer (DT) and Extended Direct Transfer (eDT) (11). Unlike conventional approaches, Robotic Extended Direct Transfer (reDT) closely resembles the eDT process. For a visual representation of the workflow, refer to Fig. 1.

The MBT Pathfinder instrument can operate with or without the Feeder attachment. When the Feeder is not attached, loading agar plates onto the MBT Pathfinder manipulation tray must be done manually by the operator. First, all agar plates are sequentially inserted into the MBT Pathfinder, and images of the agar plates are taken under six different lighting conditions that capture different visual aspects of the colonies. The software then presents these images to the operator for inspection. The software automatically detects and pre-selects circular isolated colonies as the best candidates for transfer, but the operator can modify the colony selection based on their experience or remove the plate from the analysis altogether (due to impurities, contamination, etc.). Once the colonies are selected/approved on each dish, the operator can initiate the automated colony-picking process and leave the instrument to proceed autonomously.

During the automated colony picking process, 1 µL of 70% formic acid is deposited onto each spot on the MALDI target plate, onto which the designated colony is then smeared. Once the formic acid gets dried, 1 µL of the HCCA MALDI matrix (12) is applied and left to dry. The drying processes are expedited by warming the target plate to 50°C, enhancing the efficiency of the sample preparation procedure. Additionally, it is important to note that the transferring wire used for colony picking is sterilized after each transfer by heating it to a high temperature to prevent cross-contamination. The volumes of applied reagents are comparable to manual preparation.

A notable difference between eDT and reDT is that reDT deposits formic acid before smearing the sample onto the spot. By adding the formic acid first, the colony material immediately contacts the acid, ensuring effective lysis of microbial cells. This method also helps reduce the risk of cross-contamination between samples, as the acid starts the sterilization process at the spot before any biological material is introduced. Moreover, the presence of the formic acid allows spreading the microbial biomass onto the MALDI target plate spot more homogeneously.

The MBT Pathfinder supports two of the most common MALDI target plate solutions - reusable (MSP 96 target polished steel BC) and disposable (MTP Biotarget 96). The study was designed so that both are included in the experiments.

The instrument can process 95 agar plates (one sample from each plate) and one Bacterial Test Standard (BTS) spot (96 spots on the MALDI target plate) in 127 min, with required manual interactions by the user totaling 21 min. These manual interactions are primarily performed at the beginning and end of the workflow, allowing the operator flexibility as they do not need to be present throughout the entire process.

This study was a part of a clinical validation study of the MBT Pathfinder system. All prepared MALDI target plates thus require calibration with the Bruker IVD BTS solution. The BTS is applied to the MALDI target plate manually, followed by the subsequent application of the HCCA MALDI Matrix by the instrument.

### Prospective method comparison experiment with clinical isolates

The method comparison experiment aimed to assess the clinical performance of the MBT Pathfinder in comparison with the manual preparation of the MALDI-TOF samples. *N* = 500 clinical isolates were collected per site from microbiology laboratory routine, including gram-positive (*n* = 200, 40%) and gram-negative (*n* = 200, 40%) bacteria, anaerobes (*n* = 50, 10%) as well as yeasts (*n* = 50, 10%) with a maximum of 20 isolates per species. By this, the experiment should reflect frequent microbes occurring in day-to-day laboratory routines.

**FIG 2:**
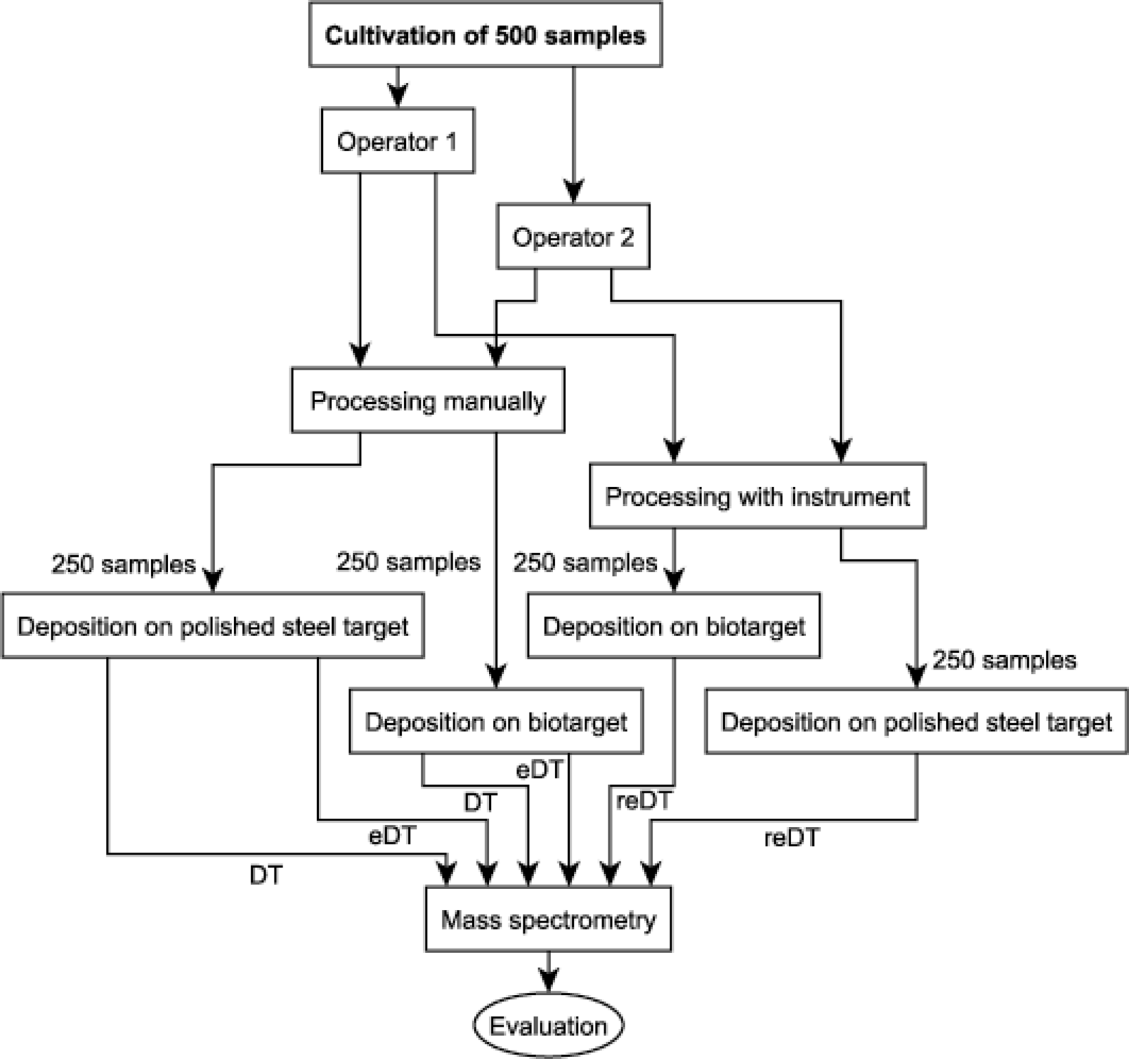
Diagram of the method comparison experiment.

All samples were prepared both manually (one spot each with DT and eDT methods) and with the MBT Pathfinder (two spots with reDT method). Every operator processed the collected 500 samples. The diagram in Fig. 2 describes the experiment workflow in the laboratory.

To evaluate the differences in performance between the methods, statistical analyses was conducted using ANOVA to determine whether there are significant differences among the methods. Post-hoc Tukey’s honestly significant difference (HSD) test was performed to identify specific differences between individual methods. This robust statistical approach ensures a comprehensive comparison of the methods’ performance.

### Repeatability experiment

**FIG 3:**
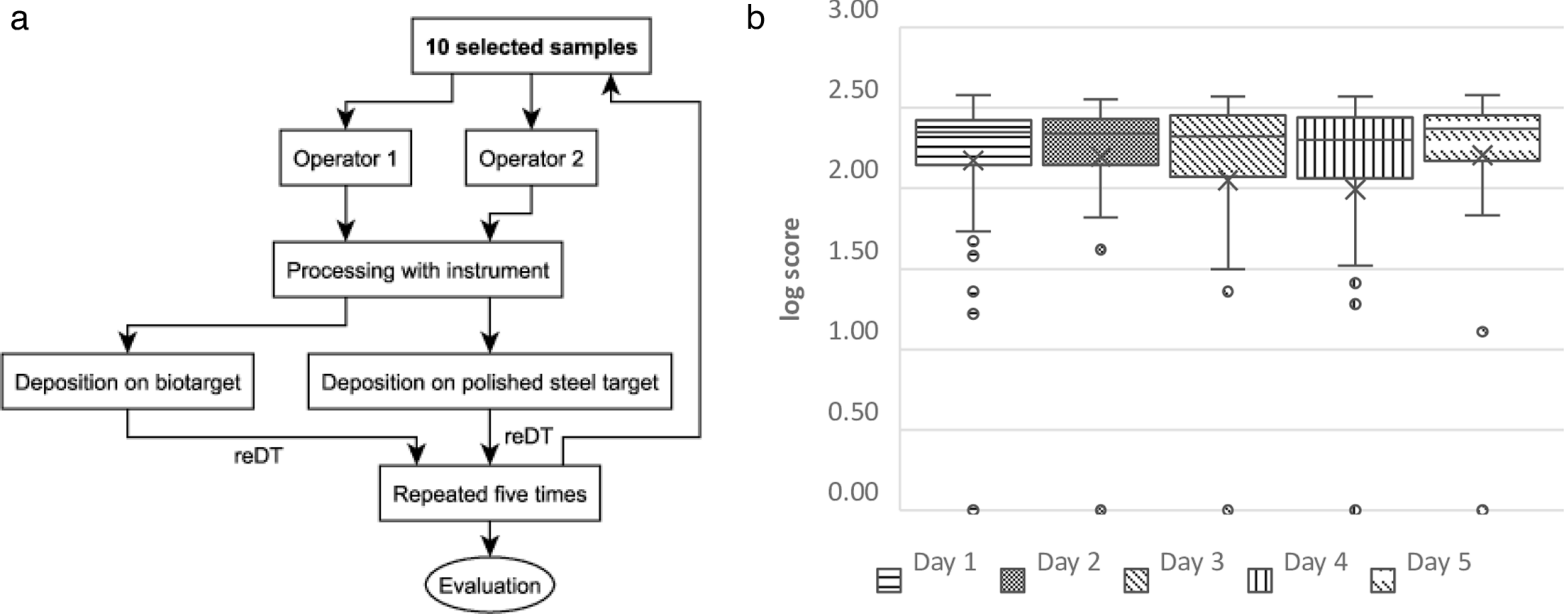
Repeatibility experiment.

To assess the repeatability of the MBT Pathfinder instrument, frequently occurring microbes cleared for identification with the MALDI Biotyper by the FDA 510(k) Premarket Notification K163536 were used (listed in Table 1). This selection ensured a representative range of commonly encountered microbes, including both aerobic and anaerobic bacteria, as well as yeasts (Fig. 3a illustrates the experiment workflow).

**TABLE 1:** List of microbes selected for the repeatability experiment

Microbial group	Growth environment	Microbial species
G-	Aerobe	*Escherichia coli*
		*Klebsiella pneumoniae*
	Anaerobe	*Pseudomonas aeruginosa*
		*Bacteroides fragilis*
G+	Aerobe	*Enterococcus faecalis*
		*Staphylococcus aureus*
		*Streptococcus pneumoniae*
	Anaerobe	*C. perfringens*
Yeasts		*Candida albicans*
		*Candida glabrata*

All samples were exclusively prepared using the automated reDT workflow. Each operator conducted two runs per day for a total of five days. The first run utilized the MSP 96 target polished steel BC, and the second run use MTP Biotarget 96. Each of the 10 samples was prepared in triplicates, with three neighboring spots on the MTP utilized for this purpose.

To evaluate the consistency of the instrument’s performance across different days, statistical analyses were conducted using ANOVA to determine if there were significant differences among the days. Post-hoc Tukey’s HSD testing was performed to identify specific differences between individual days. This statistical approach provided a detailed assessment of the instrument’s repeatability and performance stability over time.

### Challenge panel experiment

**FIG 4:**
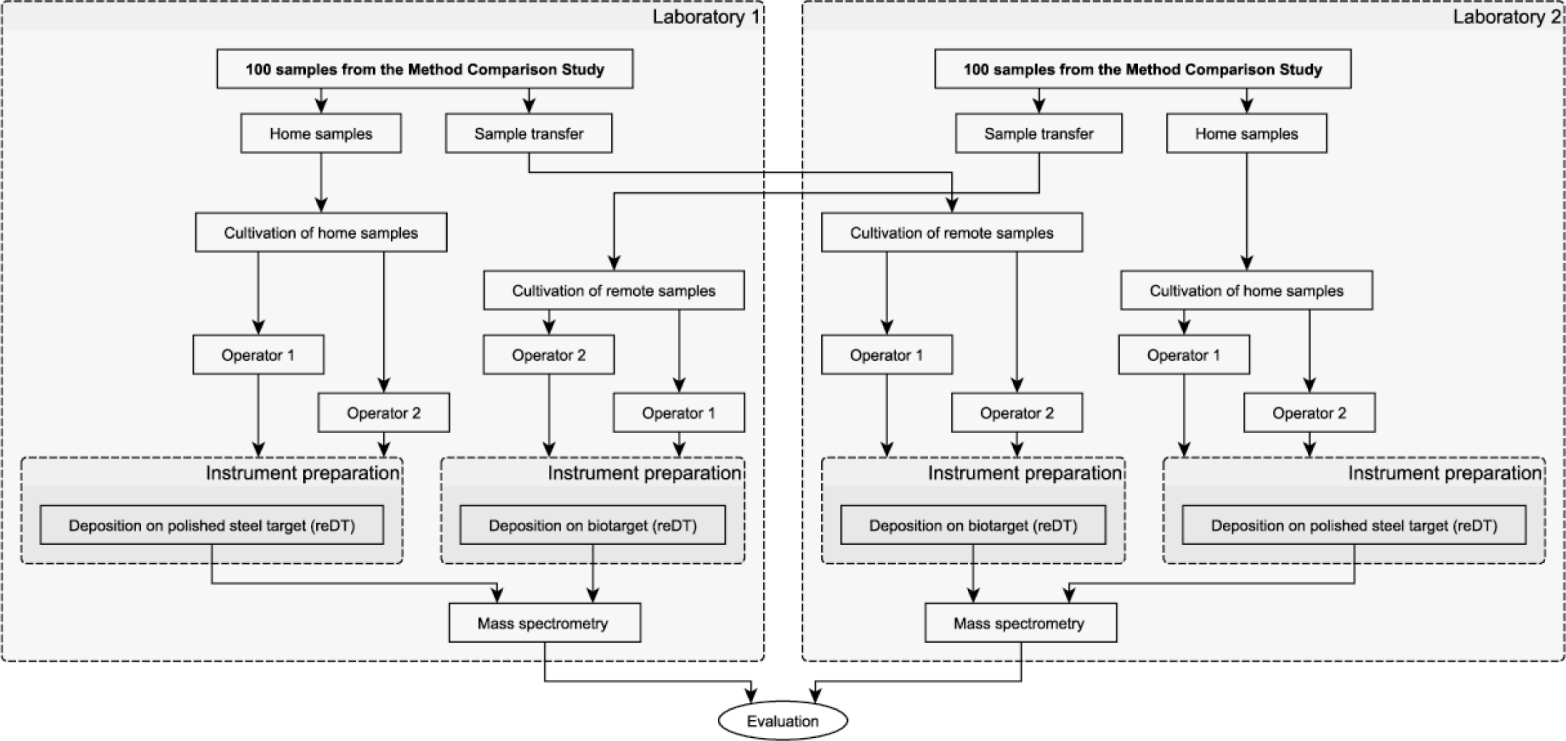
Diagram of the challenge panel experiment.

The challenge panel experiment is a comprehensive study of the instrument’s performance on commonly occurring microbes. One hundred samples from the method comparison experiment were used for this study, with all samples prepared exclusively through the automated reDT workflow. Half of each lab’s samples were set up using the disposable MALDI target plate, while the other half were shared with another laboratory where they were set up using the reusable steel MALDI target plate. The experiment workflow diagram is depicted in Fig. 4. Finally, mass spectrometry analysis and evaluation were carried out.

## RESULTS

In Table 2, a summary of results from the method comparison experiment in both laboratories is provided. The results from the automated reDT sample preparation were assessed based on strict safety requirements. The MBT Pathfinder system documents the entire colony-picking process with images. For colonies that failed during mass spectrometry, we reviewed the data trail. If any requirements were not met due to operator error, the data were excluded to ensure we evaluated only the instrument’s performance without human interference. In total, 183 samples were removed out of 4,040 samples, accounting for approximately 4.5% of the tested specimens.

**TABLE 2:** Summary of method comparison experiment results, showing average MALDI scores with standard deviations for different preparation methods across various microbial groups.

Microbe	DT	eDT	2× reDT
A	2.19 ± 0.25	2.12 ± 0.38	2.10 ± 0.43
G+	2.05 ± 0.43	2.17 ± 0.38	2.04 ± 0.55
G-	2.32 ± 0.24	2.33 ± 0.17	2.20 ± 0.40
Y	1.21 ± 0.90	1.83 ± 0.54	1.80 ± 0.56

Colonies smaller than 1 mm in diameter were excluded because the MBT Pathfinder system guarantees accuracy up to this size with its transferring wire. Examples of microbes with too small colonies that we encountered in the study include *Aerococcus urinae*, *Corynebacterium simulans*, and *Finegoldia magna*. This accounted for 65 samples (35.5%) of the exclusions. Highly mucoid bacteria were also excluded due to the risk of contaminating the instrument’s internals. During the picking process, mucoid strings can move with the transferring wire and cause contamination. Examples of highly mucoid microbes that occurred in the study include *Acinetobacter pittii* and *K. pneumoniae*. This accounted for 18 samples (9.8%) of the exclusions. However, the MBT Pathfinder can detect such strings, abort the picking process, and sterilize the wire to prevent contamination. Finally, 100 samples (54.7%) were excluded due to the operator approving the wrong position of the colony. This occurred when the operator mistakenly selected areas on the agar plate that did not correspond to the intended microbial colonies, leading to unsuccessful sample transfer and preparation. These errors highlight the importance of proper training and experience for operators to ensure accurate colony selection and minimize human error. Additionally, the sample preparation of colonies that become movable after contact with the transfer wire poses a potential limitation for the MBT Pathfinder system, though no such samples occurred in this study.

In anaerobe samples, despite a significant ANOVA result (*P* = 0.0461), post-hoc Tukey’s HSD testing revealed no significant differences between DT and eDT (*P* = 0.2409), DT and reDT (*P* = 0.1287), and eDT and reDT (*P* = 0.943). This suggests that while the overall ANOVA was significant, the differences between individual methods are not significant. For samples prepared with DT, 153 out of 182 (84.1%) had MALDI scores above the 2.0 threshold. For eDT, 150 out of 182 (82.4%) samples achieved scores above 2.0, and for reDT, 305 out of 364 (83.8%) samples exceeded the 2.0 threshold.

For G+ samples, the post-hoc Tukey’s HSD test shows that DT (2.05) was significantly different from eDT (2.17, *P* < 0.0001) and eDT was significantly different from reDT (2.04, *P* < 0.0001), but there was no significant difference between DT and reDT (*P* = 0.9175). This suggests that DT and reDT are similar in performance, while both differ significantly from eDT. Although eDT performed slightly better than reDT, the reDT method still maintained a mean score above 2.0. For DT, 533 out of 736 (72.4%) samples had MALDI scores above the 2.0 threshold. For eDT, 649 out of 736 (88.2%) samples achieved scores above 2.0, and for reDT, 1149 out of 1472 (78.1%) samples exceeded the 2.0 threshold.

In G- samples, according to the post-hoc Tukey’s HSD test, DT had a mean score of 2.32, which was significantly different from reDT’s mean score of 2.20 (*P* < 0.0001). Similarly, eDT, with a mean score of 2.33, showed a significant difference compared to reDT (*P* < 0.0001). This indicates that reDT differs significantly from both DT and eDT, while DT and eDT do not differ significantly from each other. Despite these differences, all methods achieved mean scores above 2.0, indicating generally good performance, with eDT performing slightly better than reDT. For DT, 767 out of 783 (98.0%) samples had MALDI scores above the 2.0 threshold. For eDT, 760 out of 774 (98.2%) samples achieved scores above 2.0, and for reDT, 1376 out of 1566 (87.9%) samples exceeded the 2.0 threshold.

For Yeast samples, post-hoc Tukey’s HSD testing reveals that DT (1.21) significantly differs from both eDT (1.83, *P* < 0.0001) and reDT (1.81, *P* < 0.0001), indicating that both eDT and reDT have significantly better performance than DT. However, eDT and reDT do not exhibit a significant difference (*P* = 0.9374), suggesting that although they perform significantly better than DT, their performance is comparable to each other. For DT, 48 out of 194 (24.7%) yeast samples had MALDI scores above the 2.0 threshold, while eDT achieved 90 out of 194 (46.4%) samples above 2.0, and reDT reached 193 out of 388 (49.7%) samples above 2.0. When considering a threshold of 1.7, DT had 105 out of 194 (54.1%) samples, eDT had 171 out of 194 (88.1%) samples, and reDT had 338 out of 388 (87.1%) samples exceeding this threshold.

The repeatability experiment, as detailed in Table 3 and illustrated in Fig. 3b, assessed the stability of the MALDI-TOF sample preparation process over multiple days and laboratories. The analysis utilized ANOVA with post-hoc Tukey’s HSD testing to evaluate the differences among experimental days. The results (*P* < 0.0001) revealed significant variations between certain days: day 1 and day 4 (*P* = 0.0159), day 2 and day 4 (*P* = 0.0046), and day 4 and day 5 (*P* = 0.0021). These findings suggest fluctuations in the instrument’s performance across different experimental days, with day 4 showing notable deviations compared to the others.

**TABLE 3:** Summary of repeatability experiment results, showing average scores with standard deviations to illustrate the stability of MALDI-TOF sample preparation across days and laboratories.

	Day 1	Day 2	Day 3	Day 4	Day 5	Average
Laboratory 1	2.19 ± 0.50	2.21 ± 0.46	2.24 ± 0.42	2.00 ± 0.81	2.31 ± 0.17	2.19 ± 0.47
Laboratory 2	2.15 ± 0.58	2.17 ± 0.56	1.86 ± 0.91	1.99 ± 0.76	2.10 ± 0.73	2.05 ± 0.71
Total	2.17 ± 0.54	2.19 ± 0.51	2.05 ± 0.67	1.99 ± 0.78	2.21 ± 0.45	2.12 ± 0.59

With the exception of day 4, where the average score dipped to 1.99, the scores across all laboratories consistently exceeded 2.0, indicating effective microbe identification at the species level. Notably, the first laboratory consistently achieved higher identification scores, especially evident on the third day, while the second laboratory had more identifications with zero scores. Despite the fluctuations, the average scores across laboratories remained consistently higher than 2.0, underlining the overall effectiveness of the microbe identification process.

The challenge panel experiment demonstrated the consistent and accurate preparation of commonly occurring microbial samples using the colony-picking instrument. Across both laboratories, the instrument achieved average scores ranging from 1.76 to 2.19 in this experiment, showcasing its reliable performance. The experiment also revealed that the choice of MALDI target plate type (steel or biotarget) did not significantly impact the average scores, as they were very similar (2.01 vs. 1.98). This suggests that the type of target plate may not be a critical factor influencing the instrument’s performance. Interestingly, the compiled summary in Table 4 highlights variations in performance across laboratories, with one showing higher scores with the steel plate and the other with the biotarget.

**TABLE 4:** Summary of challenge panel experiment results, showing average scores with standard deviations for samples prepared on steel and biotarget plates across two laboratories.

		Average	
		Steel	Bio
Laboratory 1	1^st^ half	1.92 ± 0.77	NA
	2^nd^ half	NA	1.76 ± 0.88
Laboratory 2	1^st^ half	2.09 ± 0.53	NA
	2^nd^ half	NA	2.19 ± 0.43

It should be noted that a score of 1.98 would not be fully reportable for many laboratories using unmodified cutoffs, which typically require scores ≥2.0 for clinical reporting. However, judging from the method comparison experiment, most samples will achieve sufficient scores, and the lower scores are more likely to be associated with challenging samples, such as yeasts. This suggests that while the instrument generally provides reliable results, additional attention may be needed for certain sample types to ensure they meet clinical reporting standards.

Additionally, we compiled a table (see Table A1 in the Appendix) of all the microbes processed by the instrument and their corresponding scores across all experiments. It provides a comprehensive overview of the instrument’s performance across a range of sample types, microbial species, and agar types, and can be used to evaluate the instrument’s effectiveness for various research and clinical applications. We excluded microbes with less than 10 samples. Furthermore, we assessed the performance of different agar types, with the average log score for BD Columbia Agar with 5% Sheep Blood being 2.17 and for BD™ Schaedler Agar with Vitamin K1 and 5% Sheep Blood being 2.19.

## DISCUSSION

The presented results illustrate the instrument’s capability to effectively prepare microbial samples, showcasing comparable performance across various types of bacteria with the samples being prepared manually by skilled laboratory technicians.

It is worth noting that the instrument’s performance on yeast samples is comparable to the manually performed eDT method because both eDT and reDT are effective in lysing the cellular wall structure during the preparation process with formic acid. The manual eDT method, while delivering superior performance, is significantly more time-consuming. In contrast, the simpler DT method is less time-consuming but shows inferior performance. The reDT method of the instrument effectively balances efficiency and high performance, being fundamentally based on the enhanced eDT preparation method.

In the repeatability experiment, a notable trend emerged where the first laboratory consistently achieved higher identification scores, especially on the third day (with average scores of 2.24 and 1.86, respectively). A closer examination of the data revealed a higher incidence of zero scores in identifications on the third day in the second laboratory.

**FIG 5:**
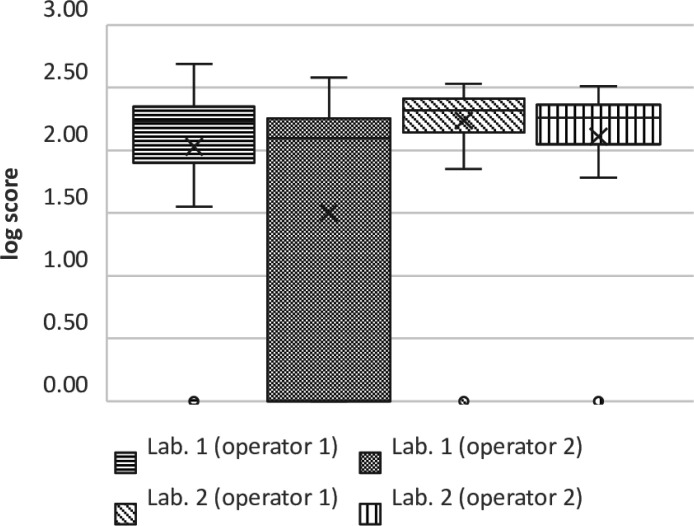
Boxplots with performance of the operators.

Furthermore, the challenge panel experiment provided additional validation of the instrument’s reliability in real-world laboratory settings. Notably, samples prepared by one operator consistently exhibited lower performance, as shown in Fig. 5. This discrepancy underscores the influence of human intervention even within an automated process and highlights the pivotal role of the operator in ensuring accurate decision-making during colony selection. Although all operators underwent the same initial training with the instrument, the operator with the lower score was less experienced, particularly with MALDI-TOF, which likely contributed to the observed differences in performance. The advantage of the reDT-method in comparison to real-word laboratory settings without automated picking is that the conditions for colony-picking are the same for each operator due to the automated presentation of the plate with an excellent camera and in optimal lighting conditions.

However, it is important to acknowledge that the instrument has certain limitations. Firstly, the instrument faces challenges in preparing samples of highly mucoid stringing bacteria due to the associated risk of cross-contamination. Secondly, sample preparation of colonies that become movable after contact with the transfer wire poses a limitation, as the wire may struggle to obtain a sufficient amount of microbial material. Lastly, the instrument is unable to obtain colonies with a diameter smaller than 1 mm, primarily attributed to the size constraints of the transferring wire. These limitations are noteworthy considerations for users and researchers employing the instrument in various laboratory settings.

When compared to a similar system (Colibrí by COPAN Diagnostics Inc.) (13, 14), the MBT Pathfinder proves more suitable for deployment in smaller laboratories owing to its compact size. Moreover, in the context of the increasingly prominent topic of sustainability in laboratories (15, 16), the MBT Pathfinder demonstrates a strong commitment. Its innovative sterilization system, featuring two transferring wires on a single axis, minimizes plastic waste while ensuring sample integrity and result accuracy. Following sample pickup and deposition onto the target plate spot with one of the wires, the wire undergoes heating at approximately 1,000°C in a specialized chamber to prevent cross-contamination. Subsequently, the deposition process seamlessly continues with the second wire, allowing the first wire to cool down before its next use. Additionally, the support for reusable MALDI target plates and the utilization of stainless steel reagent deposition needles, rather than plastic one-time-use alternatives, further underscore its dedication to sustainable practices and responsible use.

### Conclusion

The research presented in this study offers valuable insights into the performance and potential applications of the MBT Pathfinder, an automated colony-picking robot designed to streamline sample preparation for MALDI-TOF mass spectrometry. The experiments conducted shed light on its effectiveness in handling a diverse range of microbes, including bacteria and yeasts.

Results from the method comparison experiment showcased the instrument’s comparable performance with different microbial types, demonstrating its versatility and suitability for microbiology research and diagnostics. Notably, yeast species presented a distinctive challenge due to variations in cellular wall structure, emphasizing the importance of tailored preparation methods.

The repeatability experiment highlighted the stability of the instrument’s performance over multiple days and in different laboratory settings. While automated, human oversight remains crucial for decision-making during the process, emphasizing the importance of operator training and vigilance.

The challenge panel experiment further validated the reliability and accuracy of the instrument in preparing commonly occurring microbial samples.

In conclusion, the MBT Pathfinder proves to be a promising tool in the realm of microbiology laboratories, offering automation and efficiency in sample preparation for MALDI-TOF mass spectrometry. Its potential to enhance workflow, minimize plastic waste, and provide consistent and reliable results makes it a valuable asset for modern microbiology research and clinical applications.

## APPENDIX

The compiled summary of the instrument's performance across various microbial species is shown in Table A1 below.

­

**TABLE A1:** Compiled summary of the instrument’s performance across various microbial species

Microbe	Preparation count	Score		
		≥ 2.0 (%)	< 2.0 ∧ ≥1.7 (%)	< 1.7 (%)
*Acinetobacter baumannii*	28	19 (68)	5 (18)	4 (14)
*Acinetobacter pittii*	72	47 (65)	15 (21)	10 (14)
*Acinetobacter radioresistens*	12	9 (75)	3 (25)	0 (0)
*Acinetobacter ursingii*	44	31 (70)	10 (23)	3 (7)
*Aerococcus urinae*	23	14 (61)	8 (35)	1 (4)
*Alcaligenes faecalis*	12	10 (83)	2 (17)	0 (0)
*Bacteroides fragilis*	254	248 (98)	5 (2)	1 (0)
*Bacteroides ovatus*	49	39 (80)	10 (20)	0 (0)
*Bacteroides thetaiotaomicron*	65	58 (89)	7 (11)	0 (0)
*Bacteroides vulgatus*	24	22 (92)	2 (8)	0 (0)
*Candida albicans*	296	187 (63)	107 (36)	2 (1)
*Candida dubliniensis*	15	4 (27)	10 (67)	1 (7)
*Candida glabrata*	157	117 (75)	38 (24)	2 (1)
*Candida krusei*	14	8 (57)	4 (29)	2 (14)
*Candida parapsilosis*	40	23 (58)	17 (43)	0 (0)
*Candida tropicalis*	58	15 (26)	41 (71)	2 (3)
*Citrobacter freundii*	42	42 (100)	0 (0)	0 (0)
*Citrobacter koseri*	104	103 (99)	1 (1)	0 (0)
*Clostridium perfringens*	146	142 (97)	2 (1)	2 (1)
*Corynebacterium amycolatum*	55	43 (78)	9 (16)	3 (5)
*Corynebacterium simulans*	11	9 (82)	2 (18)	0 (0)
*Corynebacterium striatum*	82	77 (94)	4 (5)	1 (1)
*Enterobacter bugandensis*	18	18 (100)	0 (0)	0 (0)
*Enterobacter cloacae*	160	140 (88)	19 (12)	1 (1)
*Enterococcus avium*	11	10 (91)	0 (0)	1 (9)
*Enterococcus casseliflavus*	12	12 (100)	0 (0)	0 (0)
*Enterococcus faecalis*	281	266 (95)	12 (4)	3 (1)
*Enterococcus faecium*	141	130 (92)	11 (8)	0 (0)
*Escherichia coli*	328	324 (99)	4 (1)	0 (0)
*Klebsiella aerogenes*	53	51 (96)	2 (4)	0 (0)
*Klebsiella oxytoca*	186	163 (88)	19 (10)	4 (2)
*Klebsiella pneumoniae*	283	261 (92)	22 (8)	0 (0)
*Klebsiella variicola*	30	25 (83)	4 (13)	1 (3)
*Lactobacillus gasseri*	17	15 (88)	2 (12)	0 (0)
*Morganella morganii*	128	125 (98)	3 (2)	0 (0)
*Prevotella bivia*	30	27 (90)	2 (7)	1 (3)
*Proteus mirabilis*	160	156 (98)	4 (3)	0 (0)
*Proteus vulgaris*	14	12 (86)	2 (14)	0 (0)
*Providencia rettgeri*	20	20 (100)	0 (0)	0 (0)
*Pseudomonas aeruginosa*	279	272 (97)	7 (3)	0 (0)
*Raoultella ornithinolytica*	20	17 (85)	1 (5)	2 (10)
*Serratia marcescens*	57	52 (91)	5 (9)	0 (0)
*Staphylococcus aureus*	300	299 (100)	1 (0)	0 (0)
*Staphylococcus capitis*	19	13 (68)	6 (32)	0 (0)
*Staphylococcus caprae*	23	20 (87)	2 (9)	1 (4)
*Staphylococcus epidermidis*	135	115 (85)	18 (13)	2 (1)
*Staphylococcus haemolyticus*	106	82 (77)	23 (22)	1 (1)
*Staphylococcus hominis*	41	38 (93)	3 (7)	0 (0)
*Staphylococcus lugdunensis*	35	32 (91)	3 (9)	0 (0)
*Staphylococcus saprophyticus*	27	14 (52)	13 (48)	0 (0)
*Staphylococcus simulans*	63	58 (92)	5 (8)	0 (0)
*Stenotrophomonas maltophilia*	59	46 (78)	12 (20)	1 (2)
*Streptococcus agalactiae*	163	134 (82)	26 (16)	3 (2)
*Streptococcus anginosus*	36	33 (92)	3 (8)	0 (0)
*Streptococcus dysgalactiae*	31	19 (61)	9 (29)	3 (10)
*Streptococcus gallolyticus*	13	7 (54)	6 (46)	0 (0)
*Streptococcus oralis*	22	15 (68)	5 (23)	2 (9)
*Streptococcus pneumoniae*	103	93 (90)	8 (8)	2 (2)
*Streptococcus pyogenes*	16	13 (81)	3 (19)	0 (0)
*Streptococcus salivarius*	12	7 (58)	5 (42)	0 (00)
